# Investigating the relationship between euthanasia and/or assisted suicide and rates of non-assisted suicide: systematic review

**DOI:** 10.1192/bjo.2022.71

**Published:** 2022-06-03

**Authors:** Anne M. Doherty, Caitlyn J. Axe, David A. Jones

**Affiliations:** School of Medicine, University College Dublin, Ireland; School of Bioethics, University of Washington, Washington, USA; Department of Bioethics, St Mary's University Twickenham, UK

**Keywords:** Psychiatry and law, suicide, mortality, ethics, human rights

## Abstract

**Background:**

Euthanasia and assisted suicide (EAS) are practices that aim to alleviate the suffering of people with life-limiting illnesses, but are controversial. One area of debate is the relationship between EAS and suicide rates in the population, where there have been claims that availability of EAS will reduce the number of self-initiated deaths (EAS and suicide combined). Others claim that legislation for EAS makes it acceptable to end one's own life, a message at variance with that of suicide prevention campaigns.

**Aims:**

To examine the relationship between the introduction of EAS and rates of non-assisted suicide and self-initiated death.

**Method:**

We conducted a systematic review to examine the association between EAS and rates of non-assisted suicide and of self-initiated death. We searched PubMed, Scopus, PsycINFO and Science Direct, until 20 December 2021. Studies that examined EAS and reported data on population-based suicide rates were included.

**Results:**

Six studies met the inclusion criteria; four reported increases in overall rates of self-initiated death and, in some cases, increased non-assisted suicide. This increase in non-assisted suicide was mostly non-significant when sociodemographic factors were controlled for. Studies from Switzerland and Oregon reported elevated rates of self-initiated death among older women, consistent with higher rates of depressive illnesses in this population.

**Conclusions:**

The findings of this review do not support the hypothesis that introducing EAS reduces rates of non-assisted suicide. The disproportionate impact on older women indicates unmet suicide prevention needs in this population.

The practices of a healthcare professional ending the life of a patient on request (euthanasia), and of a healthcare professional supplying a patient with the means to end their life (assisted suicide), have been proposed since the late 19th century as means to alleviate the suffering of people with life-limiting illnesses. However, it was not until the end of the 20th century that euthanasia and assisted suicide (EAS) became established in The Netherlands, and assisted suicide (but not euthanasia) became established in Switzerland and in the state of Oregon in the USA. In the 21st century other jurisdictions, including Canada, Columbia, New Zealand and the states of Victoria and Western Victoria in Australia, have adopted similar proposals variously termed ‘medical assistance in dying’, ‘medical aid in dying’, ‘physician aid in dying’, ‘voluntary assisted dying’, ‘physician-assisted dying’ or, more generally, ‘assisted dying’. In recent years, it has been argued that the term ‘assisted suicide’ is misleading because ‘the practice of physician aid in dying is distinct from the behaviour that has been traditionally and ordinarily described as “suicide”’.^1^ Clearly it is possible to make a distinction between these practices. For example, it is possible to give separate estimates for rates of death under the provisions of ‘physician aid in dying’ legislation and rates of death by suicide in the general population. The question is whether the conceptual or empirical differences between these practices are such as to make the language of ‘assisted suicide’ inapplicable or inappropriate. This is highly contested.^2–5^

Different terminal conditions are associated with EAS: one study based in The Netherlands reported that the most common diagnosis across the regions was cancer.^6^ Data from Belgium shows that rates of EAS for dementia have increased over time.^7^ In some jurisdictions, EAS for primary psychiatric indications is permitted. These include The Netherlands, Belgium and Switzerland; Canada has recently passed legislation to permit EAS where there is no terminal illness, from 2023.^8^ The criteria can be challenging to establish where the primary condition the person is seeking relief from is a mental illness, and research has recently attempted to identify how the criteria of ‘irremediable suffering’ is met.^9^

A recent systematic review of the characteristics of EAS for psychiatric indications suggested that the characteristics of people who die by EAS for mental health reasons are very similar to the characteristics of people who die by ‘traditional’ suicide, with high numbers having psychiatric disorders and a history of self-harm.^10^ This review indicated that the demographics of the two groups have key differences, with EAS being more common in women, and traditional suicide more common in men. Suicidal ideation and behaviours may be associated with a range of mental illnesses and conditions, including psychosis, depression, personality disorders and even adjustment disorders.^11,12^ Depression and suicidal thoughts are not uncommon in end-of-life care, and there are guidelines for the management of depression in palliative care that build on the evidence base for treatment in this area.^13^ At the very least, there are similarities between suicide in the general population and cases of EAS for mental health reasons or for cases of physical health with concomitant mental illness.

## Terminology and rationale

This paper uses EAS as an umbrella term to refer to euthanasia and/or assisted suicide. The paper uses the term ‘non-assisted suicide’ for suicide that occurs in the general population without assistance of a medical kind, i.e. suicide that is not EAS. This paper uses ‘self-initiated death’ to cover death by EAS and/or by non-assisted suicide.

Although EAS has been legalised in several countries in the past 20 years, it remains subject to intense debate. One area of contention is the relationship between EAS and rates of suicide (either including or not including assisted suicide). Some proponents have claimed EAS will help reduce suicide, paradoxically, as people will feel comforted by the knowledge that there is an option for them to leave their suffering, should it become unbearable.^14,15^ They suggest that introducing EAS will reduce the number of self-initiated deaths or, even if self-initiated deaths increase, non-assisted suicides will be reduced, as people will be more likely to choose a death in a medically supported setting than a potentially violent death by suicide. Prompted by this, England's Health Secretary requested data on suicide rates among terminally ill people in April 2021, to provide evidence for the debate on the legalisation of EAS in England and Wales.^16^ Conversely, opponents of EAS have made the claim that making EAS available will not only increase the rate of self-initiated deaths, it will likely increase, or at least not decrease, rates of non-assisted suicide. The argument is that by making EAS legally available, society is making it acceptable to intentionally end one's own life, a message which is at variance with, and may undermine the consistent messaging of, suicide prevention groups and campaigns.^17^

## Aims

In this review we examine the relationship between the introduction of EAS and rates of non-assisted suicide and self-initiated death. In particular, the review seeks to assess whether there is evidence of an association, either positive or negative, between the introduction of EAS and rates of non-assisted suicide, or between the introduction of EAS and rates of self-initiated death.

## Method

This study is a systematic review of the association between EAS and suicide. It was based on the Preferred Reporting Items for Systematic Review and Meta-Analysis (PRISMA) guidelines for systemic review and meta-analysis. We did not require ethical approval as the study involved secondary analysis of anonymised data. A protocol was pre-registered with PROSPERO (identifier CRD42021277581).

To allow for a robust systematic review of the relationship between EAS and self-initiated death and/or non-assisted suicide, we conducted a search of the following databases: PubMed, Scopus, PsycINFO and Science Direct. We included all articles from the beginning of records until 2021. We restricted the search to those studies published in the English language and in peer-reviewed journals. We included original research papers only, and did not include case reports or series, or systematic or narrative reviews.

We used the following search terms: (euthanasia[title] OR assisted[title] OR ‘assistance in’[title] OR ‘aid in’[title]) AND (suicid*[title]). The searches were conducted on 21 December 2021. We based our search terms on the review conducted by Calati et al.^10^ A hand search was conducted of the reference list of all studies selected for full review.

We included studies that focused on EAS and reported data on population-based suicide rates. Where the title suggested a paper suitable for inclusion we examined the abstract, and following this, where both title and abstract suggested an article eligible for inclusion, the full article was examined for suitability for inclusion based on the inclusion criteria. The citations of all included studies were hand-searched for any additional studies meeting the inclusion criteria. Any discrepancies between the two reviewers (C.J.A. and A.M.D.) were resolved by consultation with the third author (D.A.J.). Abstracts that did not refer to both EAS and rates of non-assisted suicide were excluded. Three reviewers (C.J.A., A.M.D. and D.A.J.) examined the full texts and graded them independently.

### Quality assessment

A quality assessment was performed. We used two established assessments for assessing the quality of papers for inclusion: the Oxford quality assessment and the Newcastle–Ottawa Scale (NOS).

The NOS was used to assess the quality of non-randomised controlled studies.^18^ The NOS is a reliable tool for appraising the methodological quality of research.^19,20^ The NOS contains eight items, which are categorised into three dimensions: the selection, comparability and the outcomes of studies. The NOS ranges from zero to nine stars, as follows: selection of the study group (up to four stars/points), comparability of cohorts (up to two stars/points) and ascertainment of outcome (up to three stars/points). High-quality studies achieve more than seven stars, medium-quality studies between four and six stars and poor-quality studies achieve fewer than four stars.

## Results

**Fig. 1:**
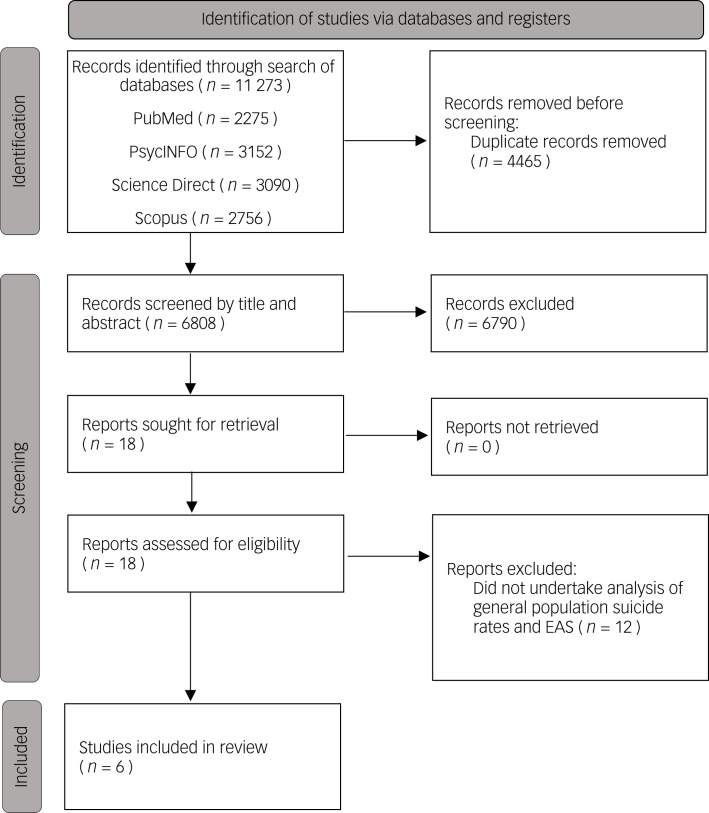
Preferred Reporting Items for Systematic Review and Meta-Analysis (PRISMA) flow diagram for selection of studies in the systematic review. EAS, euthanasia and assisted suicide.

The combined search strategies yielded a total of 11 273 titles to be screened, and 6808 remained after the removal of duplicates (Fig. 1). Following review of titles, 199 remained. In most cases where titles were excluded, this was on the basis that the term ‘suicide’ occurred in the title only in the pairing ‘assisted suicide’ and there was no indication of relevance to non-assisted suicide. After examination of title and abstracts, 18 remained, and these studies were reviewed in full. No further studies were added following a hand search of the references of the included studies.

As all of these studies were retrospective and conducted at a population level, the level of detail regarding individuals was limited. We found six studies that met our inclusion criteria. The main details around methodology and primary findings are outlined in Table 1.

**Table 1:** Characteristics and summary of the included studies examining population suicide rates and EAS

Study	Study design	Sample	Methods	Conclusions
Canetto and McIntosh, 2021^26^	Cohort study	Individuals dying by suicide and EAS between 1998 and 2018 over the age of 18 years in Oregon state (USA)	DWDA and suicide death rates per 100 000 calculated using WISQARS and WONDER data from the CDC and OHA annual reports for Oregon state and the USA	For women over 75 years of age, EAS rates are higher than non-assisted suicide rates. For women over 65 years of age, non-assisted suicides have increased in the past two decades. Women over 65 years of age represent 16% of non-assisted suicide deaths but 46% of deaths by EAS
Jones and Paton, 2015^22^	Cohort study	Individuals dying by suicide and EAS in Oregon, Washington, Montana, Vermont and the USA broadly, from 1990–2013	Used non-assisted suicide rates from 1990–2013 based on age group and EAS data from Oregon and Washington. Controlled for suicide-related demographic variables, and used logistic regression to control for confounding variables	When state effects are controlled for, rates of self-initiated death increased by 8.9% where EAS is legal. When demographic variables are controlled for, self-initiated death rates increased by 11.8% where EAS is legal. Accounting for state-specific time trends, the increase in self-initiated death is 6.3%. There are higher rates in people aged over 65 years. There is no evidence to suggest a decrease in non-assisted suicide rates or increase in mean age of death by suicide
Nanner, 2021^25^	Cohort study	Belgian individuals who died by suicide between 1990 and 2015	Used the synthetic control method to observe changes in non-assisted suicide rates in Belgium before and after legalisation of EAS (2002) compared with countries without EAS policy. Control for variables that affect suicide risk	GSCM showed an average annual suicide rate increase of 0.73 per 100 000 population (95% CI −5.7 to 7.2; *P* = 0.80). Placebo testing based on the SCM analysis showed equal outcomes for Belgium and the comparisons. This study failed to show a statistically significant association between the legalisation of EAS and non-assisted suicide rates
Steck et al, 2015^23^	Longitudinal cohort study	8 527 786 Swiss men and women with a total of 24 842 suicides between 1991 and 2008	Used the Swiss national cohort to calculate rates of EAS and non-assisted suicide (by various methods) from 1991–2008. Compared genders and age groups (15–34, 35–64 and 65–94 years), using coding provided by Federal Statistical Office	Across all age groups, increase in female self-initiated death from 13.6 to 14.3 between 1991 and 2008. In those aged over 65 years, suicide by poisoning rates doubled in men and more than tripled in women. Rate of suspected EAS increased from 2.37 to 14.98
Steck et al, 2016^24^	Longitudinal cohort study	5 million Swiss 2003 to 2008	Data on EAS from 2003–2008 provided by right-to-die organisations. Calculated rates of EAS and non-assisted suicides as associated with demographic and socioeconomic variables. Compared percentages of self-initiated death with different underlying causes. Logistic regression analysis performed to examine gender differences in probability of suicide based on gender	Rates increase with age, with a greater increase for EAS than non-assisted suicide (13.8 to 30.1 *v*. 0.3 to 38.9). Rates of EAS similar in men and women (4.1 *v*. 5.0); however, men have a non-assisted suicide rate three times that of women
Zalman and Stack, 1996^21^	Population based time series analysis	All deaths by suicide in The Netherlands from 1950–1990, using 1973 and 1981 as key legal markers	National suicide rates were obtained from the national database. Specifically rates for age groups 65–74 and ≥75 years were calculated with data from the World Health Organization. Rates were compared before and after two major cases in 1973 and 1981. This study controlled for divorce rate, religiosity and economic strain. Yule Walker techniques were used to purge for autocorrelation	Legal changes surrounding euthanasia had a significant association with non-assisted suicide rates on bivariate analysis. However, once divorce, religiosity and economic strain were controlled for, the association was no longer statistically significant, neither in the population as a whole or among older people (aged 65–74 and over 75 years)

EAS, Euthanasia and Assisted Suicide; DWDA, Death with Dignity Act; WISQARS, Web-based Injury Statistics Query and Reporting System; WONDER, Wide Ranging Online Data for Epidemiologic Research; CDC, Center for Disease Control; OHA, Oregon Heath Authority; GSCM, Generalised synthetic control method; SCM, synthetic control method.

The earliest study that sought to establish the relationship between EAS and suicide was by Zalman and Stack.^21^ Published in 1996, this study aimed to examine whether legal changes around the acceptability of EAS were associated with changes in the non-assisted suicide rates (numbers of deaths by suicide per 100 000 population) in the nation as a whole. This was not legislation for EAS, but rather changes in case law, which resulted in widespread changes in availability and practice. No reliable data were available for the rates of EAS, which is a significant limitation of this analysis. There was a significant positive association between EAS and suicide rates at both time points on bivariate analysis; however, once divorce, religiosity (as measured by the sale of religious books) and economic strain (as measured by unemployment rates) were controlled for, the association was no longer statistically significant, although it remained positive. Given the size of the population, this positive change is meaningful in itself: statistical significance is used in smaller samples to estimate whether an association is the result of chance. It is worth noting that raw population suicide rates rose throughout the period of the study (i.e. through the various legislative changes examined) from approximately six per 100 000 to approximately ten per 100 000.^21^

In 2015, Jones and Paton examined EAS and non-assisted suicide rates in USA states where EAS had been legalised or decriminalised (Washington, Oregon, Vermont and Montana), and reported increased rates of both suicide and self-initiated death between 1990 and 2013.^22^ Data on rates of non-assisted suicide were obtained from the Centers for Disease Control and Prevention in all four states, and data on EAS were obtained from the regulators in Washington and Oregon; however, data on EAS rates in Montana and Vermont were not available. Rates of self-initiated death (which Jones and Paton termed ‘total suicide’) rose in all four states, and this was significant after controlling for unemployment rate, income, race and ethnicity, religious adherence, laws on cannabis use and drink driving, and state-specific trends – an increase of 6.5% in the population of the four states, and an increase of 14.5% in those aged 65 years and older.^22^ Rates of non-assisted suicide rose significantly by 4.4% after controlling for state and year effects and socioeconomic factors. However, this result was not robust to the inclusion of state-specific trends, after which the association was positive, but not statistically significant.

Also in 2015, Steck et al reported an overall reduction in suicide rates during the period of their study (1991–2008) based on the Swiss National Cohort. This reduction was broadly similar to the decline in suicide rates in European Union countries, and was associated with reduced access to lethal means (specifically firearms in the case of Switzerland) and improved access to mental healthcare.^23^ Despite this, there was a rise in the mortality rate in older people, especially women. There was a significant increase in people dying by EAS. Suicide by poisoning doubled between the years 1991–1993 and 2006–2008 in men aged 65–94 years, and tripled in women in the same age bracket, where 80% of deaths by poisoning were attributed to EAS. The paper suggests that ‘further research is needed to clarify the reasons for the tripling of rates in assisted suicides in women, and the doubling of rates in men, and to what extent this difference might reflect greater vulnerability of women compared with men’.^23^

A second study by the same authors, also from the Swiss National Cohort published the following year in the *British Journal of Psychiatry*, examined non-assisted suicide and EAS in the years 2003–2008, in a population of over 5 million people.^24^ Overall, there were 5708 deaths by non-assisted suicide and 1325 by EAS. It reported fluctuations in non-assisted suicide rates (900–983 per annum), and an increase in EAS rates from 187 in 2003 to 246 in 2008. Women were overrepresented in the EAS group compared with the non-assisted suicide group, and had higher proportions of mental illness. Living alone and having no children were associated with higher rates of non-assisted suicide and EAS. Non-assisted suicide and EAS were more prevalent in people without a religious affiliation, and higher in Protestants than in Catholics, but these differences were greater in EAS than in non-assisted suicide. Higher education was positively associated with EAS, but negatively with non-assisted suicide. The authors argued that increases in EAS changed the epidemiology of suicide in Switzerland such that ‘analyses that distinguish between assisted and unassisted suicide are required to inform preventative interventions’.^24^

In 2021, Nanner examined suicide rates in Belgium, using a synthetic control and generalised synthetic control methods (i.e. a control group was created by an algorithm) based on suicide rates in other European Union countries. The Netherlands and Switzerland were excluded from the study and although Luxembourg was included in the construction of the control, the study did not provide an analysis of the impact of EAS on non-assisted suicide rates in Luxembourg.^25^ Data on suicide rates were extracted from Organisation for Economic Co-operation and Development data, and there was no data reported on rates of EAS in Belgium. Both analyses generated positive but non-significant associations of EAS and non-assisted suicide, with generalise synthetic control methods estimating an increase in the non-assisted suicide rate of 0.73 per 100 000 population (95% CI −5.7 to 7.2).^25^

Canetto and McIntosh examined the relationship between EAS and non-assisted suicide in the state of Oregon in the USA over a 20-year period (1998–2018), following the legalisation of EAS.^26^ They calculated the mortality rates by non-assisted suicide and EAS, and reported that women aged over 65 years represented almost half of all EAS deaths, and that EAS was the most common cause of self-initiated death in older women. Over this period, rates of EAS among older women increased by 496.4%, and increased among older men by 563.6% (before the legislation, rates would have been zero, so increased rates are to be expected). In this time period, non-assisted suicide rose by 56.4% among older women, whereas it fell by 10.9% among older men. Suicide by firearms was the leading cause of self-initiated death among older men. All-cause self-initiated death rose both among older women and older men.^26^

Each of these studies was graded according to Oxford and NOS assessments (Table 2). All studies scored similarly on the Oxford quality assessment. On the NOS, three scored as high-quality studies (Jones and Paton,^22^ Steck et al^23^ and Steck et al^24^), two as medium-quality studies (Nanner,^25^ and Canetto and McIntosh,^26^ although Nanner was the stronger of the two) and one as poor-quality (Zalman and Stack^21^). Zalman and Stack^21^ suffered from lack of EAS data and lack of clarity as to whether suicide figures included EAS deaths, hence lack of case definition and no controls outside The Netherlands. Canetto and McIntosh^26^ provided data for EAS and non-assisted suicide, and stratified data by gender as well as age. However, they provided no controls for socioeconomic factors, and no estimate of significance for the changes in rates of non-assisted suicide and of self-initiated death. Nanner^25^ developed a control model based on other European countries and provided a measure of significance. However, the study was limited in that it did not consider EAS data and only included one EAS country. Jones and Paton^22^ considered the introduction of EAS in four different states at four different time points, and were able to control for time and state effects as well as socioeconomic factors and state-specific linear trends. Both studies by Steck et al^23,24^ provided a detailed comparison of characteristics of cases of EAS and non-assisted suicide by analysis of the Swiss National Cohort. This was a longitudinal study of mortality in the Swiss population, based on linkage of census data with mortality records from 1990 to 2008 and including data on more than 7 million individuals.

**Table 2:** Quality assessment of the included studies

	Oxford assessment	NOS								
		Selection				Comparability	Outcome			Total NOS
		Case definition adequate	Representativeness	Selection of controls	Definition of controls	Were one or more factors controlled for	Ascertainment of exposure	Same ascertainment exposure and controls	Non-response rate	
Canetto and McIntosh^26^	2b	*	*	0	0	0	*	0	*	4
Jones and Paton^22^	2b	*	*	*	*	**	*	0	*	8
Nanner^25^	2b	0	*	*	*	*	*	0	*	6
Steck^23^	2b	*	*	*	*	**	*	0	*	8
Steck^24^	2b	*	*	*	*	**	*	0	*	8
Zalman and Stack^21^	2b	0	0	0	0	**	*	0	0	3

NOS, Newcastle–Ottawa Scale. The NOS ranges from 0–9 stars as follows: selection of the study group (up to 4 stars/points), comparability of cohorts (up to 2 stars/points) and ascertainment of outcome (up to 3 stars/points). High-quality studies achieve more than 7 stars, medium-quality studies 4–6 stars and poor-quality studies fewer than 4 stars.

## Discussion

This systematic review found that there are few studies examining the association between EAS and rates of non-assisted suicide and/or rates of self-initiated death: only six studies met the inclusion criteria. The quality of these studies varied widely. All used different methods, thus precluding meta-analysis.

These studies cover four different jurisdictions: Switzerland, The Netherlands, Belgium and the USA. It is notable that most studies (four out of six) and all of those that scored as high quality (three), focused on Switzerland or the USA rather than on The Netherlands or Belgium. Neither of the studies from the Belgium or The Netherlands sought to analyse EAS data nor assess whether the introduction of EAS was associated with an increase in overall self-initiated death. Perhaps this is because EAS has long been an accepted part of practice in these jurisdictions.

Despite these divergences in analysis and the differences in practice between the four jurisdictions, there are some common results. No study found a negative association between EAS and non-assisted suicide. Canetto and McIntosh reported a reduction in non-assisted suicide in older men in Oregon between 1998 and 2018, but the overall non-assisted suicide rate in older people in that state increased over that period and the non-assisted suicide rate in older women increased by more than 50%.^26^ Furthermore, those studies that controlled for socioeconomic factors all found a positive association between EAS and rates of non-assisted suicide, although generally these results were not statistically significant.^21,22,25^

There is no evidence from these studies to date to support the hypothesis that EAS reduces non-assisted suicide. More high-quality research is needed before it can be determined definitively whether there is no association between introduction of EAS and rates of non-assisted suicide, or whether there might be a small positive association, as suggested by some estimates in the study by Jones and Paton.^22^

Nanner argues that, although there is no evidence that the availability of EAS prevents death by suicide, it may reduce suicidal feelings.^25^ He cites a study of 100 consecutive applications for euthanasia on the basis of a mental health in Belgium.^27^ Of these, eight were accepted but patients withdrew because ‘knowing they had the option gave them peace of mind to continue living; ergo, there was some alleviation of suicidal ideation’. However, Nanner does not acknowledge that, of these 100 patients, 43 had died by the end study period: 35 by EAS, one by palliative sedation (used in Belgium as an alternative to EAS), six by non-assisted suicide and one from anorexia nervosa; eight were still pursuing their euthanasia requests. An intervention that results in 42% of suicidal patients dying by self-initiated death over a 5-year period would not appear to be an effective means to alleviate suicidal ideation.

In relation to self-initiated deaths, Jones and Paton provide evidence for a significant increase of 6.5% in the USA, after controlling for state and year effects, socioeconomic factors and state-specific trends.^22^ The increase was 14.5% for those aged over 65 years. Steck et al^23^found that although suicide rates in Switzerland generally declined, a ‘substantial increase’ in EAS in older women resulted in ‘a net increase in the rate of suicide overall [i.e., of self-initiated death] in women’. At that time, rates of self-initiated death in men in Switzerland were decreasing but less so in older men because of EAS. Furthermore, since that time, rates of EAS have risen in all jurisdictions where it is legally available, and this has driven increases in self-initiated death.^28^ The change in rate of self-initiated death in Oregon between 1998 and 2018 for those aged over 65 years can be derived from the figures provided by Canetto and McIntosh.^26^ Over this period, self-initiated death among those aged over 65 years increased by 59.6%m with an increase in older men of 23.3% and an increase in older women of 190.2%. Nanner provides no figures for EAS. However, since the introduction of the euthanasia law in Belgium in 2002, officially reported deaths by EAS have increased consistently year on year, with 2022 deaths by EAS recorded in 2015, up from 235 deaths in 2003. Nanner reports no significant decrease in non-assisted suicide in Belgium between 2002 and 2015, which suggests a substantial increase in self-initiated death.^25^ Of these papers, only Jones and Paton provide confidence intervals for the estimated increase in self-initiated death after the introduction of EAS.^22^ Nevertheless, Canetto and McIntosh and Steck both provide evidence of large increases in EAS driving overall increases in rates of self-initiated death, and no paper provides evidence that would conflict with this conclusion.^23,24,26^

One feature that was not part of the initial research question, but has emerged from the review, is the importance of considering gender when exploring associations between EAS, rates of non-assisted suicide and rates of self-initiated death. Canetto and McIntosh and both studies by Steck et al show that the change in self-initiated death after the introduction of EAS may be far greater in older women than in older men.^23,24,26^ This is an omission in the studies by Jones and Paton, and Nanner.^22,25^ The reasons why women may be more likely to die by EAS have been outlined variously as empowerment and disempowerment, depending on the perspective of the commentators. Data from suggests that women are more likely to seek EAS, with fears of being a burden: this consideration may be a factor in the gender disparity.^26,29^ These disparities may be associated with lifelong disadvantages in social, occupational and economic areas in the context of women perceiving they are not as highly valued as men, and may have internalised a view that their value is relative to their service to others. Women also have higher incidences of depressive illnesses, which may indicate an unmet mental health need.

Several authors have drawn on this literature on suicide contagion as the basis for claims about the potential impact of EAS on non-assisted suicide rates or rates of self-initiated death.^30–32^ However, whether there is such an effect and, if so, the size of this effect and the factors that might exacerbate or mitigate it, must be established independently. This review shows that there has been little quantitative research exploring this association to date. More research is urgently required to inform the debate around the legalisation of EAS in those jurisdictions where EAS is prohibited, and to inform suicide prevention strategy in jurisdictions where EAS is legally available.

In this review, the authors did not discover any other systematic literature review considering the potential association between EAS, rates of non-assisted suicide and rates of self-initiated death. This contrasts with the related topic of the impact of media reporting of suicides on suicide rates, variously termed suicide contagion or the ‘Werther effect’. This has been the focus of many original research papers and several systematic reviews and meta-analyses.^33–36^ This evidence base has, in turn, informed the development of guidance for the media on reporting suicide, the implementation of which has itself been the subject of review.^37^

In some jurisdictions where EAS has been decriminalised, it remains an offence to counsel, incite or encourage suicide.^38^ This suggests that, even in jurisdictions where it is legal, EAS should not be promoted, and that, in parallel with efforts to prevent non-assisted suicide, consideration should be given to strategies to address the factors that make people vulnerable to death by EAS. There is a clear need for mental healthcare and suicide prevention among people with physical illnesses who would be eligible for EAS in some jurisdictions.^39,40^ This review has found that that the availability of EAS is not associated with a reduction in non-assisted suicide, and that it may result in an increase in self-initiated deaths in women. The challenge of effective suicide prevention among older adults and those with chronic or terminal illnesses remains.

Limitations of this review include the small number of studies internationally that have explored the association between EAS and suicide in the general population. The primary research is itself limited by the challenges in controlling for the multitude of factors that affect both suicide and EAS. The only study that outlined the underlying diagnosis was the 2016 Swiss-based study by Steck et al, which reported that approximately 5% of people who died by EAS had a diagnosis of a mental illness.^24^ This reflects the paucity of research in this area and indicates that there is a need to build the evidence base for better management of mental illnesses associated with suicidality, especially in people with physical (including terminal) illnesses. A further limitation is the fact that the data included in this study were all derived from the peer-reviewed literature and did not include independently produced reports. Also, this review may have missed some studies published in non-English language journals, as they were excluded from the searches. Finally, there may be a future role for a more focused systematic review to examine the effects of gender on uptake of EAS in more detail, where available.

This systematic review suggests that there is no association between EAS and reduced suicide rates in the countries where it is legislated for. This has consequences for the overall suicide prevention efforts in these and other countries, and may have particular implications for older women. The paucity of studies that could be included in this systematic review indicates a need for robust research into the covariates of suicidal ideation and self-initiated death in countries where EAS is legislated for. There is a need for specific research to examine the reasons for the disproportionate impact on the mortality of older women, and the impact of such legislation on groups with pre-existing psychiatric diagnoses or mental health problems. Further research should include consultations with expert individuals and groups in the jurisdictions where EAS is legal.

In conclusion, the findings of this review do not support the claims made that the introduction of EAS results in reduced rates of non-assisted suicide. Several studies reported increases in overall rates of self-initiated death and, in some cases, increases non-assisted suicide, although this latter increase was generally not significant when sociodemographic factors were controlled for. The studies based in Switzerland and Oregon suggest that older women might be disproportionately vulnerable to EAS where this is legislated for, and when the higher rates of depressive illnesses among women are considered, this may indicate a need to address suicide prevention more assertively in this population.

## Data availability

The data that support the findings of this study are available from the corresponding author, A.M.D., upon reasonable request.
